# Millionaires

**Published:** 1874-03

**Authors:** 


### MILLIONAIRES.

### WHERE WILL THE RICH MEN COME FROM?

WHAT becomes of the sons of our great men, is a question that is frequently asked and as frequently left unanswered. The intellectual powers of the father, if predominant, seldom descend to the son. In a certain sense this rule holds true with respect to the ability to acquire and retain riches. If the father possess this in a remarkable degree, the son, in nine cases out of ten, is a spendthrift. Examples of this are not wanting. The descendants of men who, two or three generations ago, rolled in opulence, hold clerkships or other subordinate positions. Wealth, influence and ability in some families descend from father to son; but these are isolated cases, and, as exceptions, only prove the truth of the rule. Since they are so seldom retained in one family for any great length of time, the query, where our rich men of the future will come from naturally suggests itself. They do come to the surface, and gradually unfold these powers which enable them to manage vast enterprises, control millions, and wield a mighty influence. The result is not a freak of fortune; they are not kicked into good luck. Their success is merely the result of long and laborious years—a right appreciation of the details. Wealthy young men begin life just where their fathers left off, and, of course, end where their fathers began—*i. e.*, at the little end of the horn. Our future rich men are to-day peddling fish in the streets, selling oranges or papers on the sidewalk, or are engaged in some remunerative employment, the wages of which are each week divided between current expenses and the savings bank—the latter generally getting the lion's share.

